# Developing a predictive model for early urinary incontinence after laparoscopic radical prostatectomy: a retrospective cohort study

**DOI:** 10.3389/fonc.2026.1838376

**Published:** 2026-07-07

**Authors:** Yuan Tang, Tianlu Li, Xiaoxi Song, Linghui Qin

**Affiliations:** 1 Department of Urology, The People’s Hospital of Tongnan District Chongqing City, Chongqing, China; 2 Department of Neonatology, The People’s Hospital of Tongnan District Chongqing City, Chongqing, China; 3 Department of Urology, Xiangyang Central Hospital, Affiliated Hospital of Hubei University of Arts and Science, Xiangyang, China

**Keywords:** laparoscopic surgery, predictive model, prostate cancer, prostatectomy, urinary continence

## Abstract

**Objective:**

To create a predictive model for early urinary incontinence following laparoscopic radical prostatectomy (LRP).

**Methods:**

This study examined clinical data from 408 patients who received LRP at Xiangyang Central Hospital during the period December 2020 to December 2025. Patients were divided into groups based on the presence or absence of urinary incontinence. Univariate and multivariate logistic regression analyses were conducted to identify independent predictors of urinary incontinence, which were then used to construct a nomogram. The predictive model’s discrimination, calibration, and clinical utility were assessed via receiver operating characteristic (ROC), calibration, and decision curves.

**Results:**

The analysis included 408 patients. Among the participants, 168 (41.2%) were categorized as having urinary incontinence, while 240 (58.8%) were not. Univariate logistic regression analysis identified prostate volume, membranous urethral length (MUL), intravesical prostatic protrusion length (IPPL), clinical staging and neurovascular bundle (NVB) preservation as predictors of urinary incontinence. Multivariate logistic regression analysis further confirmed that prostate volume, MUL, IPPL and NVB preservation were independent predictors. Subsequently, a clinical prediction model was developed and visualized as a nomogram, with its discriminative performance evaluated using a ROC curve. The area under the curve (AUC) was 0.910 (95%CI 0.862–0.957), demonstrating good discriminatory power. Good consistency was observed from the calibration curve, and a significant clinical net benefit was shown by the decision curve.

**Conclusions:**

The prostate volume, MUL, IPPL and NVB preservation have a good predictive value for early urinary incontinence after LRP, and the resulting nomogram demonstrates good predictive performance.

## 1 Introduction

Radical prostatectomy remains the cornerstone treatment for localized and locally advanced prostate cancer (1–3), with urinary incontinence being a prevalent postoperative complication. After radical prostatectomy, the majority of patients recover from urinary incontinence within one year, but early postoperative urinary incontinence still significantly affects the quality of life of patients (4, 5). Factors such as patient condition, surgeon experience, tumor status, and surgical technique all influence urinary incontinence following radical prostatectomy (6–8). Predicting postoperative urinary continence recovery using preoperative anatomical factors has recently gained significant attention in prostate cancer surgery research (9, 10). The anatomical factors under investigation primarily consist of parameters related to the sphincter, gland, pelvic floor support structures, and pelvis. A meta-analysis has identified membranous urethral length (MUL) as an independent risk factor for postoperative urinary continence recovery following radical prostatectomy, highlighting its significance as a sphincter-related parameter (11). Parameters related to the prostate gland—including prostate volume and intravesical prostatic protrusion length (IPPL)—that are measured using pelvic MRI are associated with surgical difficulty, complication risks, neurovascular bundle preservation, and postoperative functional outcomes (12) The urinary continence recovery prediction model aids in evaluating pre-surgical incontinence risk and informs treatment selection for prostate cancer patients. In previous studies, the prediction models for postoperative urinary continence recovery were mainly constructed based on clinical factors and questionnaire survey results of patients (13–15). This retrospective study examined postoperative urinary continence recovery in prostate cancer patients who underwent laparoscopic radical prostatectomy (LRP) at Xiangyang Central Hospital between December 2020 and December 2025. It also aimed to develop a predictive model for postoperative urinary incontinence using gland- and sphincter-related parameters obtained from MRI.

## 2 Materials and methods

### 2.1 Study design and population

We retrospectively analyzed prostate cancer patients who underwent LRP at Xiangyang Central Hospital from December 2020 to December 2025. We excluded patients with a history of prostate surgery or urinary incontinence, lack of a preoperative MRI, need for preoperative catheterization due to urinary retention, receipt of neoadjuvant endocrine therapy or radiotherapy, or incomplete clinical data. Collected clinical data included age, BMI, PSA levels, biopsy Gleason score, clinical stage, whether to preserve the neurovascular bundle (NVB), and surgeons. The final sample size was contingent upon the consecutive series of eligible patients who presented during the predefined study period.

### 2.2 MRI examination methods and data measurement

All patients underwent preoperative MRI examination within one week before prostate biopsy. The examination was performed using a Siemens 3.0T superconducting magnetic resonance imaging scanner with an abdominal phased array coil for signal reception. The scan covered the prostate and both seminal vesicles, and included axial, sagittal, and coronal fast spin-echo T2WI and axial T1WI. The MUL was assessed on coronal MRI as the distance from the prostate’s urethral tip to where the urethra enters the bulbar urethra. MRI parameters related to the gland included prostate volume and IPPL. Prostate volume was calculated by the formula prostate volume = 0.52×length×width×height. IPPL is the vertical distance measured in the sagittal plane from the apex of the prostatic tissue protruding into the bladder to the bladder base. MUL, prostate volume, and IPPL were measured by two independent radiologists. Each reader possessed over a decade of experience in diagnosing abdominal and pelvic imaging. Prior to measurement, both radiologists underwent a 3-hour pre-training session, which included practical training on interpreting and measuring MUL, prostate volume, and IPPL using 20 non-study cohort cases. During the training, they standardized the specific anatomical landmarks and procedural protocols for measuring these three parameters. Two radiologists independently and blindly reviewed all preoperative MRI images. The blinding design included the following aspects: (1) blinding to patient identity, clinical pathological information, and surgical outcomes; (2) mutual blinding between the two radiologists (i.e., no exchange of measurement results before each completed their measurements); and (3) blinding to all clinical outcomes during measurements, including postoperative urinary incontinence status at 3-month follow-up. All image measurements were performed on a dedicated picture archiving and communication system (PACS), with consistent display parameters (window width, window level, and magnification) maintained across all measurement rounds for each case. To assess the consistency of MUL, prostate volume, and IPPL measurements, we conducted inter-observer reliability analyses. Inter-observer reliability was evaluated by randomly selecting 100 patients, whose measurements were independently performed by two radiologists, with Intraclass Correlation Coefficient (ICC) values calculated accordingly. The ICC was computed using a two-way random effects model with absolute agreement and single measurement, and results were reported as ICC values, 95% confidence intervals, and P-values (16). ICC interpretation followed these criteria: values of 0.90 or higher indicated excellent performance, 0.75 to 0.90 good performance, 0.50 to 0.75 moderate performance, and below 0.50 poor performance (17). We observed inter-observer ICCs of 0.86 (95% CI: 0.78–0.92, P<0.001) for MUL, 0.90 (95% CI: 0.84–0.95, P<0.001) for prostate volume, and 0.82 (95% CI: 0.72–0.89, P<0.001) for IPPL, indicating good inter-observer consistency across all three measures. We calculated the average of the two radiologists’ measurements to minimize random measurement error and enhance the robustness of our predictive modeling (18). In this study, the preservation of NVB during LRP was uniformly classified according to standard urological surgical techniques, with a binary classification defined as follows: 1) NVB preservation group: patients underwent unilateral or bilateral preservation of the NVB during surgery, ensuring complete protection of the nerve-vascular structures on both sides of the prostate responsible for erectile function and urinary control; 2) Non-preservation group: due to tumor factors such as extracapsular extension or high-risk surgical margins, the NVB were completely resected unilaterally or bilaterally without any preservation attempt. Group assignment was recorded by the operating surgeon intraoperatively and later verified by two independent reviewers based on surgical records postoperatively, ensuring no missing cases. All surgeries in this study were performed by two senior urologists certified in LRP. Cases were categorized strictly according to the primary surgeon listed in the surgical record, with no instances of joint primary surgeons, resulting in clear and unambiguous group assignments without ambiguous samples.

### 2.3 Surgical method

Post-anesthesia, the patient was positioned supine with an elevated waist and head lower than the feet. A puncture device was inserted at the incision below the umbilicus to establish pneumoperitoneum, with three additional devices placed at the outer edges of the left and right rectus abdominis and the inner side of the right anterior superior iliac spine. The anterior and lateral bladder tissues were dissected to expose the prostate’s ventral surface and the pelvic fascia. Following the incision of the pelvic fascia, the prostate was dissected from its lateral edge to the apex, and the deep dorsal vein complex of the penis was sutured and ligated. After incising the anterior bladder neck wall at the bladder neck-prostate junction, the urinary catheter was removed and suspended on the abdominal wall to elevate the prostate base. The prostate was then dissected from the bladder by opening the posterior bladder neck wall along the prostate. The vas deferens were bilaterally exposed and severed, after which the seminal vesicles were separated. The Denonvillier fascia was then incised between the seminal vesicles and extended to the prostate apex. In cases where preoperative assessment and intraoperative exploration indicated the possibility of preserving the neurovascular bundle, it was carefully dissected and preserved along the posterior lateral side of the prostate capsule using scissors. The apex of the prostate underwent blunt dissection to preserve the membranous urethra as much as possible. The urethra was transected to fully excise the prostate. If necessary, the bladder neck was reconstructed, and the bladder-urethral anastomosis was performed with a 5/8 arc single-needle continuous suture. A urinary catheter was retained following the anastomosis.

### 2.4 Evaluation of urinary control function after surgery

This study defines urinary incontinence as “any symptom of involuntary urine leakage,” following the recommendation of the International Continence Society (ICS) (19). The definition was operationalized using a composite criterion: (a) use of ≥1 pad per day (excluding safety pads), and/or (b) urine loss of ≥2 grams during a 24-hour pad weight test (20). The time window for “early” incontinence was defined as 3 months after LRP, consistent with previous predictive model studies (21).

### 2.5 Statistical analysis

Statistical analyses were conducted using SPSS (v25.0, IBM Corp.) and R (v4.4.1, R Foundation for Statistical Computing). Model construction and validation were conducted using relevant R packages (rms, pROC, rmda). Normality of continuous variables was evaluated using the Shapiro-Wilk test. Normality of continuous variables was evaluated using the Shapiro-Wilk test. Variables following a normal distribution were reported as mean ± standard deviation (SD) and compared between groups via the independent samples t-test. For variables not normally distributed, data were presented as median with interquartile range (IQR) and analyzed using the Mann-Whitney U test. Categorical variables were summarized as frequencies and percentages, and group differences were assessed using the Chi-square test or Fisher’s exact test, as appropriate, depending on suitability. Potential associations with urinary incontinence were initially evaluated using univariate logistic regression. Variables showing a statistically significant association (P < 0.05) in the univariate analysis were selected for inclusion in a subsequent multivariate logistic regression model. A backward stepwise selection process (removal criterion: P > 0.10) was used on the multivariate model to identify independent predictors and address multicollinearity. An individualized prediction model, presented as a nomogram, was then created using the final independent predictors from the multivariate model. The nomogram’s discriminative power was assessed using a ROC curve and AUC. The nomogram model was verified by the Bootstrap resampling method and the calibration curve was plotted to evaluate the robustness of the model. The model’s clinical benefit was evaluated using the clinical decision curve (DCA), with statistical significance determined by a two-sided P-value below 0.05.

## 3 Results

### 3.1 Selection process

**Figure 1:**
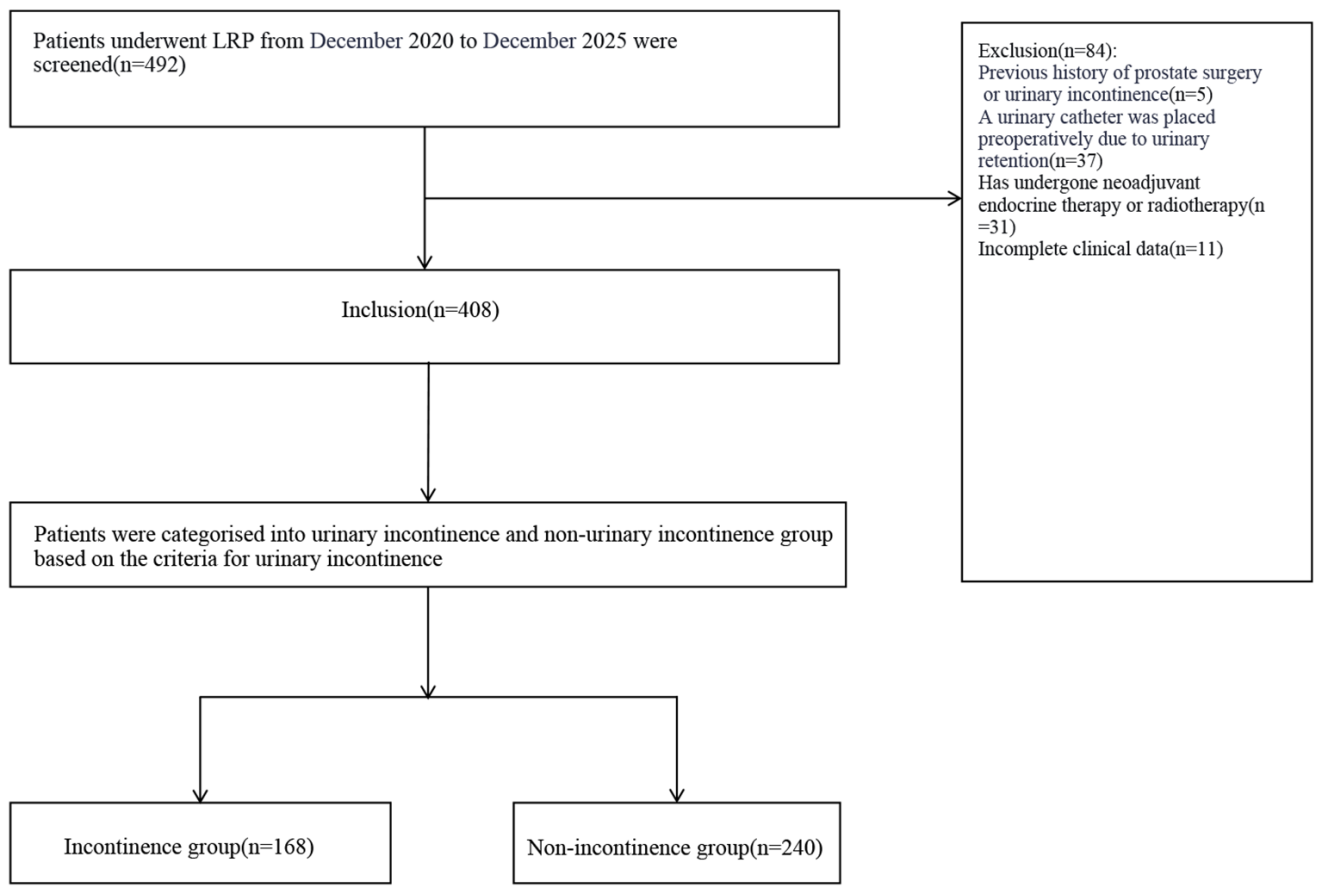
Flowchart of the screening process. LRP, laparoscopic radical prostatectomy.

The analysis included 408 patients. Of these, 168(41.2%) were classified into the urinary incontinence group and 240(58.8%) into the non-urinary incontinence group, based on the diagnostic criteria. **Figure 1** provides an overview of the patient selection process.

### 3.2 Clinical characteristics

Statistically significant differences were observed between the urinary incontinence and non-urinary incontinence groups regarding prostate volume, MUL, IPPL, clinical staging and NVB preservation (**Table 1**).

**Table 1:** Clinical characteristics of the urinary incontinence group versus the non-urinary incontinence group.

Characteristic	Incontinence N = 168	Non-incontinence N = 240	p-value
Age, Median (Q1, Q3)	69.00 (65.00, 71.00)	70.00 (66.00, 72.00)	0.328
BMI, kg/m^2^, Median (Q1, Q3)	24.60 (24.20, 25.35)	24.30 (23.75, 25.30)	0.102
PSA, Median (Q1, Q3)	14.35 (9.66, 20.59)	13.98 (11.73, 15.89)	0.862
ProstateVolume, Median (Q1, Q3)	42.00 (33.00, 55.00)	34.00 (31.00, 38.50)	<0.001
MUL, Median (Q1, Q3)	11.00 (7.00, 14.00)	15.00 (14.00, 16.00)	<0.001
IPPL, Median (Q1, Q3)	6.00 (3.00, 8.00)	1.50 (0.00, 4.00)	<0.001
GleasonScore≥7, n (%)	100 (59.5%)	148 (61.7%)	0.827
Clinical Staging, n (%)			0.001
T1/T2	44 (26.2%)	134(58.3%)	
T3	124 (73.8%)	106 (41.7%)	
NVB preservation, n (%)	13 (7.7%)	52 (21.7%)	<0.001
Surgeon			0.067
Zhou Fei(n=285)	109(38.2%)	176(62.8%)	
Sun Xiaosong(n=123)	59(48.0%)	64(52.0%)	

BMI, Body Mass Index; PSA, Prostate Specific Antigen; MUL, membranous urethral length; IPPL, intravesical prostatic protrusion length; NVB, Neurovascular Bundle.

### 3.3 Univariate logistic regression

Univariate logistic regression analysis identified prostate volume, MUL, IPPL, clinical staging and NVB preservation as predictive factors for urinary incontinence (**Table 2**).

**Table 2:** Univariate logistic regression analysis of urinary incontinence.

Variable	OR (95%CI)	P value
Age	0.944 (0.848, 1.050)	0.292
BMI	1.024 (0.932, 1.291)	0.276
PSA	1.047 (0.961, 1.144)	0.294
ProstateVolume	1.110 (1.060, 1.174)	<0.001
MUL	0.590 (0.452, 0.719)	<0.001
IPPL	1.657 (1.374, 2.092)	<0.001
GleasonScore	0.914 (0.408, 2.060)	0.827
Clinical Staging	3.945 (1.710, 9.610)	0.002
NVB preservation	0.303 (0.159, 0.577)	<0.001
Surgeon	0.672 (0.438, 1.029)	0.067

CI, Confidence Interval; OR, Odds Ratio; BMI, Body Mass Index; PSA, Prostate Specific Antigen; MUL, membranous urethral length; IPPL, intravesical prostatic protrusion length; NVB, Neurovascular Bundle.

### 3.4 Multivariate logistic regression

These significant variables were entered into the multivariate logistic regression. The findings indicated that prostate volume, MUL, IPPL and NVB preservation independently predicted urinary incontinence (**Table 3**). Univariate analysis revealed a marginal difference in early postoperative urinary incontinence between the two surgeons (P = 0.067). After adjustment for age, BMI, PSA, prostate volume, MUL, IPPL, Gleason score, clinical staging and NVB preservation, surgeon was not independently associated with incontinence (adjusted−OR = 1.12, 95%CI: 0.70–1.79, P = 0.642). Accordingly, surgeon was excluded from the final prediction model.

**Table 3:** Multivariate logistic regression analysis of urinary incontinence.

Variable	OR (95%CI)	P value
ProstateVolume	1.527 (1.332, 1.639)	0.018
MUL	0.563 (0.445, 0.681)	<0.001
IPPL	1.879 (1.248, 2.887)	<0.001
NVB preservation	0.168 (0.083, 0.236)	<0.001

CI, Confidence Interval; OR, Odds Ratio; MUL, membranous urethral length; IPPL, intravesical prostatic protrusion length; NVB, Neurovascular Bundle.

### 3.5 Nomogram

**Figure 2:**
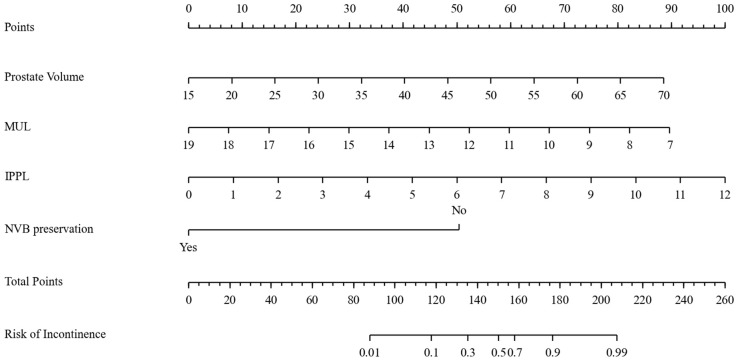
Nomogram prediction model for urinary incontinence. MUL, membranous urethral length; IPPL, intravesical prostatic protrusion length; NVB, Neurovascular bundle.

A clinical prediction model was created using independent predictors from the multivariate analysis and is illustrated as a nomogram (**Figure 2**).

### 3.6 ROC curve, calibration curve, and clinical decision curve

**Figure 3:**
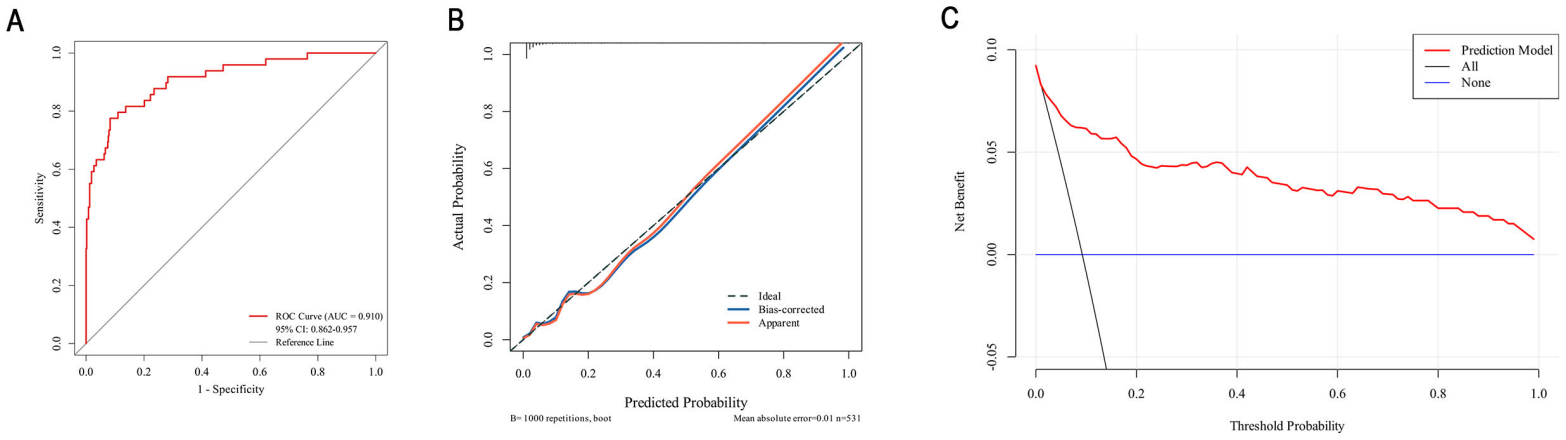
**(A)** ROC curve; **(B)** calibration curve; **(C)** DCA curve.

The model’s discriminative performance was assessed with an ROC curve. The AUC was 0.910 (95%CI 0.862–0.957), demonstrating good discrimination (**Figure 3A**). The nomogram was validated through the calibration curve, which maintained a good consistency with the standard curve, indicating a relatively robust model (**Figure 3B**). After bootstrap optimism correction, the AUC shifted marginally from 0.910 to 0.904, while the calibration slope adjusted from 1 to 0.978. The corrected model also yielded a Brier score of 0.058, a calibration intercept of -0.036, an R² of 0.271, an ICI (Eavg) of 0.007, and an Emax of 0.033. Collectively, these metrics indicate strong discrimination and calibration with a minimal overfitting risk. DCA analysis indicated the model’s substantial net benefit and considerable clinical application value (**Figure 3C**).

## 4 Discussion

Urinary incontinence frequently occurs following radical prostatectomy due to surgical impairment of the external urethral sphincter and its innervating nerves, which impacts blood supply and leads to postoperative bladder instability (22, 23). Pelvic MRI examination can provide a deep understanding of the bladder, prostate, posterior urethra and surrounding structures of prostate cancer patients before radical prostatectomy. Previous studies have confirmed that the anatomical parameters indicated by pelvic MRI examination are correlated with the recovery of postoperative urinary control function (24–27). The prostate gland’s size, shape, and its anatomical relationship with the bladder influence various surgical techniques in radical prostatectomy (28, 29). Patients with large prostates and a median lobe extending into the bladder face higher risks of perioperative complications and delayed urinary control recovery. This study examined the clinical and imaging data of 408 patients who underwent LRP surgery, focusing on postoperative urinary control recovery. Utilizing MRI-derived gland-related parameters, a statistical model was developed to predict urinary control recovery post-surgery.

This study identified prostate volume, MUL, IPPL and NVB preservation as independent risk factors for urinary continence recovery three months post-LRP. MUL is defined as the span from the prostatic urethral apex to the urethral entry point into the cavernous body. A reduced preoperative MUL has been consistently recognized in clinical studies and meta-analyses as an independent predictor of urinary incontinence after radical prostatectomy (11, 30, 31). Maximizing MUL preservation and maintaining urethral functional length during surgery, without compromising tumor removal, are crucial for enhancing postoperative urinary continence recovery (32, 33). Patients with a shorter preoperative MUL should be thoroughly informed about the risk of delayed urinary continence recovery post-operation. Bladder neck reconstruction during surgery can help mitigate the adverse effects of a shorter MUL on urinary continence recovery.

Prostate volume is the most commonly used glandular parameter related to perioperative prognosis and functional prognosis. An increased prostate volume adversely affects the preservation of neurovascular bundles, functional urethral length, and bladder-urethral anastomosis. Patients with larger prostate volume often have chronic urinary tract obstruction. If the median lobe hyperplasia is more obvious, the surgical process is also relatively complex. As the prostate enlarges, the tension of the anastomosis between the bladder and the urethra increases, making the anastomosis more difficult. The postoperative urinary control function of the patient is affected, and the risk of urinary incontinence is higher (34–36).

Prostate protrusion into the bladder is a difficult situation faced during LRP (37). The degree of prostate protrusion into the bladder can be quantitatively evaluated by measuring IPPL through preoperative MRI. This study corroborates previous findings that patients with a preoperative IPPL of ≥ 5 mm experience delayed urinary control recovery post-surgery (38).

The NVB is not merely a nerve bundle, but a composite structure composed of nerves, blood vessels, and surrounding fascia. Preserving the NVB maintains the anatomical continuity of the pelvic fascia and the lateral prostate fascia, stabilizes the pelvic floor support system, and prevents postoperative displacement of the membranous urethra and collapse of pelvic floor tissues. Its accompanying microvasculature also ensures blood supply to the urethral sphincter, reduces postoperative tissue edema and ischemic injury, and accelerates recovery of urinary control function (39, 40).

This study enhances previous nomograms for predicting urinary continence recovery post-radical prostatectomy by integrating gland-related parameters from preoperative MRI, thereby improving the accuracy of postoperative urinary incontinence risk assessment. The gland-related parameter model for predicting urinary continence recovery post-LRP is useful for preoperative consultation and patient education in prostate cancer surgery. By assessing the risk of postoperative urinary incontinence in prostate cancer patients, this model facilitates refinements in surgical techniques and postoperative rehabilitation.

Our clinical data were obtained from a Tertiary Grade A Hospital. We hypothesize that our findings are externally applicable to regions with similar economic levels. However, several limitations of this study should be noted: (1) The statistical model relies on retrospective data from a single center, and non-single-surgeon clinical data without external validation, potentially affecting its reliability; (2) Differences in surgical technique and experience may affect urinary control after LRP. After adjustment for age, BMI, PSA, prostate volume, MUL, IPPL, Gleason score, clinical staging and NVB preservation, surgeon was not independently associated with incontinence (adjusted−OR = 1.12, 95%CI: 0.70–1.79, P = 0.642). These findings indicate that the marginal crude difference in incontinence rates between the two surgeons was mainly driven by baseline patient heterogeneity rather than differences in surgical technique or experience. Both surgeons are experienced urologists (>20 years) performing laparoscopic radical prostatectomy at the same institution, which may explain the absence of significant differences in urinary control. However, this conclusion does not imply that surgical skill and experience have no impact on the outcome of urinary incontinence. This study was unable to quantify the relationship between surgical experience and outcomes, nor did it analyze the interaction between surgical experience and predictor variables; (3) The absence of objective urodynamic data limits the evaluation of postoperative urinary incontinence mechanisms and the relationship between urinary continence recovery and anatomical parameters, which will be addressed in future research.

In conclusion, prostate volume, MUL, IPPL and NVB preservation independently contribute to early urinary incontinence following LRP. The MRI gland-related parameter-based nomogram effectively predicts early urinary control recovery post-LRP. Larger-scale clinical research is necessary to validate the findings of this study.

## Data Availability

The original contributions presented in the study are included in the article/supplementary material. Further inquiries can be directed to the corresponding author.
